# Phenotype, origin and estimated prevalence of a common long QT syndrome mutation: a clinical, genealogical and molecular genetics study including Swedish R518X/*KCNQ1* families

**DOI:** 10.1186/1471-2261-14-22

**Published:** 2014-02-19

**Authors:** Annika Winbo, Eva-Lena Stattin, Charlotte Nordin, Ulla-Britt Diamant, Johan Persson, Steen M Jensen, Annika Rydberg

**Affiliations:** 1 Department of Clinical Sciences, Pediatrics, Umeå University, Umeå, Sweden; 2 Department of Medical Biosciences, Medical and Clinical Genetics, Umeå University, Umeå, Sweden; 3 Department of Public Health and Clinical Medicine, Heart Centre, Umeå University, Umeå, Sweden

**Keywords:** Long QT Syndrome, Genotype-phenotype correlations, Clinical phenotype, Founder mutation, Mutation age, Prevalence estimate

## Abstract

**Background:**

The R518X/*KCNQ1* mutation is a common cause of autosomal recessive (Jervell and Lange Nielsen Syndrome- JLNS) and autosomal dominant long QT syndrome (LQTS) worldwide. In Sweden p.R518X accounts for the majority of JLNS cases and is the second most common cause of LQTS. Here we investigate the clinical phenotype and origin of Swedish carriers of the p.R518X mutation.

**Methods:**

The study included 19 Swedish p.R518X index families, ascertained by molecular genetics methods (101 mutation-carriers, whereof 15 JLNS cases and 86 LQTS cases). In all families analyses included assessment of clinical data (symptoms, medications and manually measured electrocardiograms), genealogy (census records), haplotype (microsatellite markers) as well as assessment of mutation age and associated prevalence (ESTIAGE and DMLE computer software).

**Results:**

Clinical phenotype ranged from expectedly severe in JLNS to surprisingly benign in LQTS (QTc 576 ± 61 ms vs. 462 ± 34 ms, cumulative incidence of (aborted) cardiac arrest 47% vs. 1%, annual non-medicated incidence rate (aborted) cardiac arrest 4% vs. 0.04%).

A common northern origin was found for 1701/1929 ancestors born 1650-1950. Historical geographical clustering in the coastal area of the Pite River valley was shown. A shared haplotype spanning the *KCNQ1* gene was seen in 17/19 families. Mutation age was estimated to 28 generations (95% CI 19;41). A high prevalence of Swedish p.R518X heterozygotes was suggested (~1:2000-4000).

**Conclusions:**

R518X/*KCNQ1* occurs as a common founder mutation in Sweden and is associated with an unexpectedly benign phenotype in heterozygous carriers.

## Background

Loss-of-function mutations in the *KCNQ1* gene cause both the autosomal recessive Jervell and Lange-Nielsen syndrome (JLNS) and the autosomal dominant type 1 long QT syndrome (LQTS), also known as the Romano-Ward syndrome [1]. The *KCNQ1* gene encodes the α-subunit of a voltage-gated potassium ion channel (Kv7.1) regulating both inner ear endolymph flow and cardiac action potential duration via the delayed rectifier potassium current (I_Ks_). Kv7.1 function loss corresponds to congenital hearing loss in JLNS, as well as variable QT prolongation and propensity for arrhythmia, presenting as syncope or (aborted) cardiac arrest, in both syndromes.

Several hundred different mutations with variable effect on Kv7.1 function have been reported, and while mutation-specific risk-stratification could be of considerable clinical importance in LQTS, few mutations are common enough to allow such characterization [2].

In Sweden, two *KCNQ1* mutations dominate the mutation spectrum regarding LQTS and JLNS [3,4]. The p.R518X and p.Y111C mutations account for over 25% of Swedish LQTS index cases with identified mutations [3], and p.R518X alone has been identified as the major cause of JLNS in Sweden [4]. Regarding p.Y111C, strong founder effects during the population development of a northern river valley region have previously been shown to result in the enrichment of this specific founder mutation in the population [5,6].

Here we investigate the clinical phenotype and founder nature of the R518X/*KCNQ1* mutation, a worldwide known hotspot mutation and a common cause of JLNS and LQTS [2,7-9], in the Swedish population.

## Methods

### Case ascertainment

**Figure 1:**
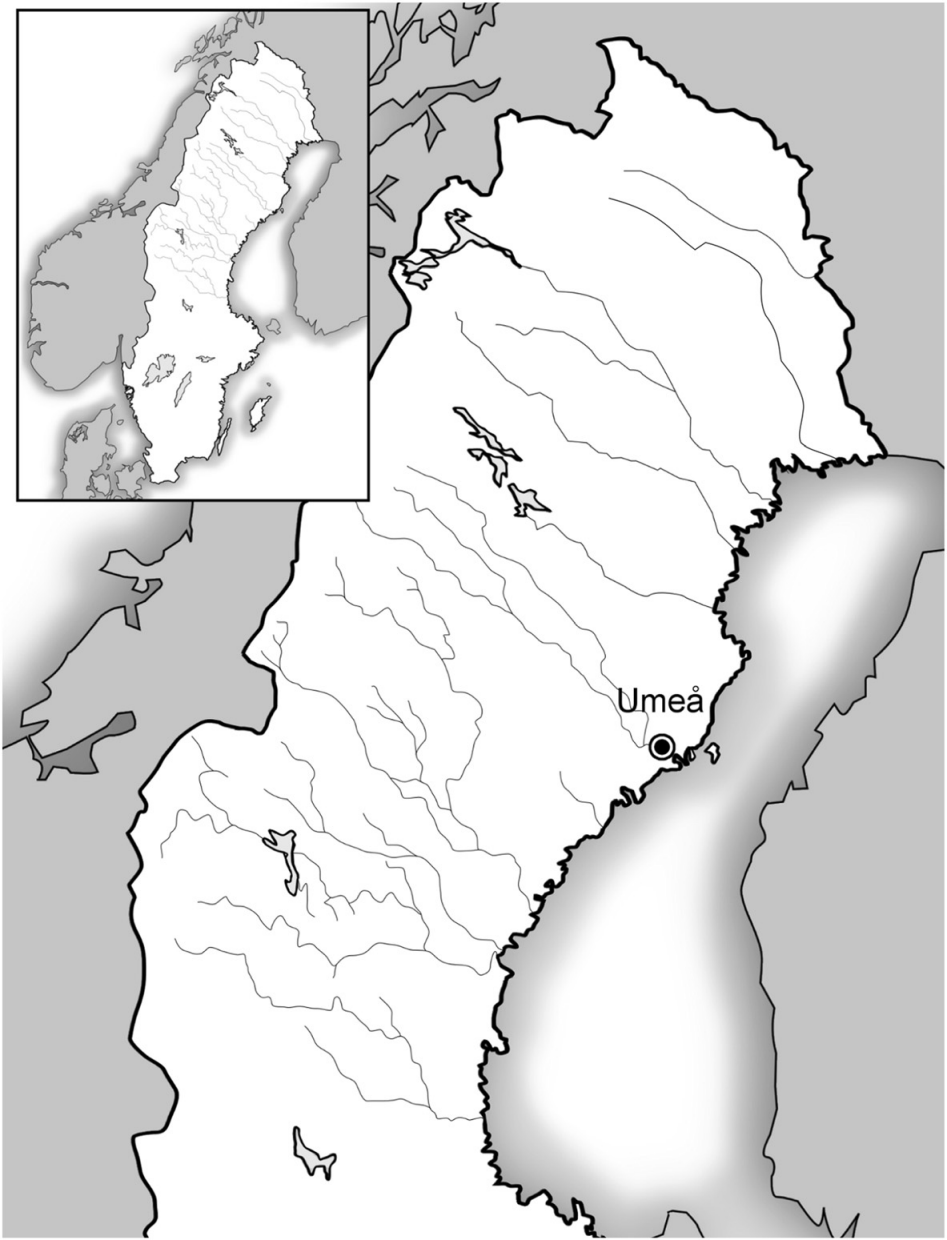
**Geographic location of Umeå situated in northern Sweden, Scandinavia (upper left corner, bordering on Norway to the west and the Gulf of Bothnia and Finland to the east).** Rivers in the northern region are depicted as lines.

Index families (n = 19) carrying the p.R518X mutation [c.1522C > T] were identified between 2001 and 2011 through the regional LQTS Family Clinic, Centre for Cardiovascular Genetics and/or national clinical referrals to the department of Clinical Genetics, both at Umeå University Hospital, Umeå, Sweden (Figure 1). Case ascertainment was performed using genomic DNA extracted by a standard salting-out procedure. Genotypes in p.R518X probands (10 LQTS index cases and 9 JLNS index cases) were ascertained by denaturing high-performance liquid chromatography (Wave 3500 HT, Transgenomic, Inc, Omaha, Neb) and/or sequencing all coding exons of the *KCNQ1* gene (CEQ 8000, Beckman Coulter, Fullerton, CA, USA), according to current clinical practices for molecular genetics diagnostics. The presence of possible additional mutations in the other major LQTS genes (*KCNH2*, *SCN5A* and *KCNE1*) was subsequently investigated and negated in 6/10 LQTS probands. Genotype was ascertained in all 9 probands of JLNS genotype, whereof all carried double *KCNQ1* mutations (including three of homozygous p.R518X genotype (p.R518X/p.R518X) and 6 of compound heterozygous genotype (p.R518X and p.M159 splice error [c.477 + 1G > A], p.R190W [c.568C > T], p.Q530X [c.1588C > T], p.S349W [c.1046C > G], or p.S227del [c.828_830del]). In family members (n = 164) mutation carrier-ship was ascertained by sequencing or targeted mutation analysis of the identified mutation (MGB-probes by ABI 7000, Applied Biosystems, Foster City, CA, USA). Index families comprised the first ascertained index case/proband in a family without known relations to any other LQTS family, plus all tested family members identified through the process of cascade-screening of first-grade relatives [5]. All p.R518X index families identified during the time period 2001-2011 were invited to participate in the study. Participants, or their legal guardian, signed an informed consent and the study was approved by the Regional Ethical Committee in Umeå, Umeå University, Sweden.

### Clinical phenotype

All living p.R518X mutation-carriers (n = 97) answered a questionnaire regarding personal LQTS history (occurrence of symptoms and beta-blocker therapy duration and compliance). Symptomatic cases were interviewed by one of the authors regarding symptoms (debut, type, frequency and triggers). Anamnestic data regarding four deceased JLNS siblings was obtained during an interview with their living sibling. The data included information regarding deafness, cardiac symptoms (debut, type and frequency) and age at, and cause of, death.

Symptomatic LQTS was defined as syncope/transient but complete loss of consciousness. Life-threatening cardiac events were defined as aborted cardiac arrest requiring resuscitation or as sudden cardiac death.

QT interval duration was measured manually, preferably in lead II as a mean of three consecutive QT intervals, and corrected for heart rate by Bazett’s formula (QT/√R-R), using the mean of the R-R intervals (lead III) preceding the measured beats, in standard 12- lead electrocardiograms obtained from medical records, when available recorded in absence of beta-blocker therapy. Beta-blocker therapy was not discontinued in any mutation-carrier in order to obtain a recording in absence of therapy.

### Statistical analyses regarding clinical phenotype

Clinical parameters were explored within p.R518X genotype groups using Chi-square test (Fisher’s Exact test, for correlations between nominal variables), and Nonparametric test (Mann Whitney U test, for analysis of variance between continuous (scalar) and nominal variables). To assess the interfamilial variation of phenotype, the annual incidence rate of life threatening cardiac events in absence of beta-blocker therapy in p.R518X heterozygotes was compared to that of a large mixed LQT1 population [10], by Fisher’s Exact test. For clinical parameters a two-tailed value for p < 0.05 was considered statistically significant.

### Investigating the origin of the p.R518X mutation

Genealogical investigations were performed using parish records and genealogical databases at the Umeå University research archive and the Swedish archive information homepage (http://www.svar.ra.se). Both parental lineages were investigated at least up until 1750, and when possible traced back to the 16^th^ century. Birth-places of ancestors born between 1650 and 1950 were noted on regional maps to assess geographical clustering, over time.

**Figure 2:**
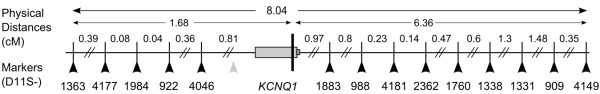
**Overview of the chosen microsatellite markers used for haplotype analysis, including 14 microsatellite markers (nomenclature D11S- followed by the number under the arrows) flanking the *KCNQ1 *gene, spanning over a distance of ~8 cM (8 × 10**^**6 **^**base pairs).** The p.R518X mutation location in *KCNQ1* is indicated by the vertical black line. Physical distances between the p.R518X mutation and each marker are given above. The grey arrowhead indicates the location of the D11S-1318 marker included in the p.Y111C haplotype analysis that was excluded in the current analysis due to a generally low quality of peaks and a high frequency of background noise.

A haplotype analysis, using 14 microsatellite markers flanking the *KCNQ1* gene (Figure 2), spanning over a distance of approximately 8 × 10^6^ base pairs, was performed in all 19 p.R518X index families and 168 control chromosomes from healthy military recruits of northern Swedish origin [5]. Haplotype analysis was preferentially performed in mutation-carriers pertaining to two separate generations in each index family (two carriers in LQTS/compound heterozygous JLNS index families and three carriers in homozygous JLNS index families) in order to aid in resolving the mutation-associated haplotype. The allele, for each marker, shared by both carriers was interpreted as the mutation-associated allele. In cases where two different alleles were present in both generations, the pattern from all included index families was used to reconstruct the most likely founder/ancestral haplotype. The control data was used to assess whether the observed pattern could be due to random chance. Fragment analysis was performed according to the manufacturers’ instructions (forward and reverse primers were provided by Sigma-Aldrich Inc.) and the PCR product analyzed using an automated capillary electrophoresis based DNA Sequencer (Wave® 3500 HT, Transgenomic Inc, Omaha, Nebraska). Solutions and material for PCR mix were manufactured by GE Healthcare (United Kingdom) and Applied Biosystems (Foster City, California). The microsatellite data was analyzed using GeneMapper software version 3.7 (Applied Biosystems, Foster City, CA, USA).

### Mutation age and prevalence estimations

The age of a mutation can be inferred by assessing the frequency of an allele, or the decay secondary to mutations and/or recombination of an ancestral haplotype over the generations in a sample of probands sharing mutations identical by descent [11,12]. In p.R518X index families sharing a common haplotype, the distance (in generations) from the included probands to the most recent common ancestor (an approximation of mutation age) as well as the associated prevalence of founder descendants (as a function of mutation age) were estimated, including 95% confidence intervals (95% CI), using the ESTIAGE software [13] and DMLE freeware (available at http://www. dmle.org).

The mutation age estimate, as calculated by the ESTIAGE software, included haplotype data, allele frequencies from healthy controls, and recombination frequencies for the microsatellite markers. The *haplotype data* included the extent of shared alleles among probands, counting from the gene and outwards, excluding shared alleles distant of any marker with discordant alleles. The *allele frequencies from healthy controls* included the proportion of the founder allele, per marker, in a sample of 168 control chromosomes of northern Swedish origin. The *recombination frequencies* were derived from the physical distances between the mutation and the microsatellite markers, calculated using the standard correspondence 1 cM = 10^6^ base pairs. Separate estimates were performed in order to assess the potential impact of the assumed mutation rate (10^-6^ to 10^-4^) as well as the mutation model used (stepwise or equal).

The approximation of p.R518X prevalence as a function of mutation age, as calculated by the DMLE software, included haplotype data from families sharing a common haplotype, regional population growth rates and an estimate of the proportion of population sampled. The *haplotype data* included the full haplotype of both p.R518X families and controls for all analysed markers. The *regional population growth rate* was analysed as a discrete variable = e^(ln [end population/start population]/number of generations) -1), calculated using population demographics data available from Statistics Sweden (http://www.scb.se). The *proportion of population sampled* was viewed as the unknown variable and iterations were performed over the interval 0.0001-0.5, within a range of possible values for mutation age (defined as the overlap between the 95% confidence intervals of the ESTIAGE and DMLE mutation age estimates). The upper limit of acceptable values for the variable proportion of population sampled was corrected for the number of ascertained mutation-carriers in the population.

## Results

### Study population and p.R518X-associated clinical phenotype

Cascade-screening in 19 Swedish p.R518X Swedish index families identified 97 p.R518X mutation-carriers (including probands) and 73 non-carriers. Among the ascertained mutation-carriers, 11 were JLNS cases (4 homozygous, 7 compound heterozygous) and 86 were heterozygous LQTS cases. Additionally, 4 deceased JLNS cases with available anamnestic and clinical data, siblings to a JLNS case with ascertained homozygous p.R518X genotype, were included in the study. Characteristics of the p.R518X study population, stratified by genotype (JLNS/LQTS), are summarized in Table 1.

**Table 1:** **Clinical characteristics of the Swedish R518X/ *KCNQ1 *** **study population**

	**JLNS**^**a,b**^	**LQTS**^**a**^
Cases	15	86
Females	8 (53)	54 (63)
Age at last follow-up, years	29 ± 23, 28	34 ± 21, 37
Non-medicated follow-up, years	16 ± 20, 9	31 ± 21, 33
Experience of first cardiac event	12 (80)	15 (17)
Age at onset, years	2 ± 1, 3	18 ± 15, 12
Experience of ACA/SCD	7 (47)	1 (1)
Number of events	9	1
Non-medicated life-years, n	241	2466
Annual incidence rate before therapy,%^c^	4	0.04
Triggers of symptoms,%		
Exercise/Swimming/Emotions/Other	46/ 12/ 2/ 20	44/ 2/ 22/ 32
ECG (% recorded off therapy)	11^d^ (27)	81 (73)
QTc, ms^e^	576 ± 61, 560	462 ± 34, 459
<440 ms	0 (0)	20 (25)
≥500 ms	10 (91)	13 (17)
Heart rate, bpm	77 ± 23, 75	73 ± 20, 69
Beta-blocker therapy	11^f^ (73)	37 (43)
Age at therapy start, years	8 ± 15, 2^g^	25 ± 19, 17

Abbreviations: JLNS- Jervell and Lange-Nielsen syndrome, LQTS- long QT syndrome, ACA- aborted cardiac arrest requiring resuscitation (not including device therapy), SCD- sudden cardiac death.

^a^ Values are expressed as number of patients (percentage of total), or as mean ± standard deviation, followed by median, if not otherwise specified.

^b^ Clinical data on JLNS cases have been previously described together with all identified Swedish JLNS cases, by our group [4]. JLNS cases in family JLN5 have been described previously by others, [14] our study is based on contact with the proband in 2010.

^c^ Calculated as number of life-threatening events/sum of non-medicated life years (×100).

^d^ Electrocardiograms were not available from the four deceased JLNS cases in family JLN5.

^e^ Intra-observer measurement error 0.4 ± 5.7 ms between repeated measurements, coefficient of variation 0.1%.

^f^ All now living JLNS cases were on beta-blocker therapy.

^g^ Among JLNS cases born from 1980 and onwards (n=8), 3 received beta-blockers during the first year of life and the remaining 5 received beta-blockers during the first 3 years of life.

JLNS cases (n = 15, QTc 576 ± 61 ms, range 461-697 ms) presented with congenital hearing loss and a severe cardiac phenotype (early symptoms debut and a high frequency of life-threatening cardiac events, including three sudden deaths, Table 1). A more severe phenotype for homozygous as compared to compound heterozygous JLNS cases was suggested in this limited material, but did not reach statistical significance (syncope 100% vs. 57%, p = 0.077; QTc 622 ± 64 ms vs. 550 ± 45 ms, p = 0.089).

LQTS cases (n = 86, QTc 462 ± 34 ms, range 397-545 ms) presented with a relatively benign overall phenotype (Table 1). Fifteen LQTS cases (17%) had experience of syncope during a mean follow up of 31 ± 21 years (median 33) before therapy. One case died suddenly and there were no aborted cardiac arrests reported (cumulative incidence of life threatening events 1.2%, annual incidence rate before therapy 0.04%). This corresponds to a low incidence of life-threatening cardiac events, as compared to a large LQT1 population [10] (0.04% vs. 0.3%, p = 0.007). The sudden cardiac death occurred in a previously asymptomatic adult female in family JLN2 (age 56 years) with a QTc of 506 ms without prophylactic beta-blocker therapy, in relation to physical exercise and hypokalaemia (probably diet-induced).

QTc in LQTS cases showed variability between males and females (443 ± 29 ms vs. 473 ± 33, p < 0.001) as well as within genders (range 397-521 ms in males; 402-545 ms in females). Based on age- and gender adjusted QTc levels [15], 46% of LQTS cases had a prolonged QTc while 54% had a borderline (28%) or normal QTc (27%). Among LQTS males, the corresponding proportions were 34%, 28% and 38%. Among symptomatic LQTS cases (n = 15), QTc ranged between 402-545 ms and 33% (five cases, whereof two males) had a QTc below 440 ms. In symptomatic LQTS cases (n = 15, whereof five males) phenotypic variability was evident regarding age at onset (1.5-56 years), cardiac event frequency (1- >10) and QTc (402-545 ms). Five symptomatic cases (33%) had a QTc below 440 ms (whereof two males).

No significant indicators of risk for cardiac events were found when assessing the distribution of the following variables across the categories symptomatic and asymptomatic heterozygous p.R518X carriers; QTc ≥500 ms (p = 0.061), gender (p = 0.56), heart rate (p = 0.112) and scalar QTc prolongation (p = 0.622).

Symptomatic LQTS cases (n = 15) were equally found in families with JLNS and LQTS probands (7 vs. 8), i.e. several LQTS probands were investigated for other reasons than previous experience of syncope, such as palpitations, dizziness or chance findings of a prolonged QTc on the electrocardiogram.

With regards to therapeutic interventions, these were performed earlier and more frequently in JLNS cases as compared to LQTS cases. Four JLNS cases (all born before 1950) died prior to beta-blocker therapy, at the age of 20, 27, 37 and 59 years, respectively. Among the 11 JLNS cases treated with beta-blockers, two cases were treated with left cardiac sympathetic denervation (age 2, 24 years) and four cases, including the previous two, received implantable cardioverter defibrillators (age 5, 10, 19 and 32 years), due to recurrent syncope in spite of therapy. All cases with implantable cardioverter defibrillators (n = 4) have experienced appropriate shocks, according to their medical records. In LQTS cases beta-blocker therapy (43% of cases, Table 1) was associated with only one case experiencing a first syncope while on therapy, and no recurrences in 11 previously symptomatic cases with adequate dosage, while compliant.

### A common origin established using genealogy and haplotype analysis

**Figure 3:**
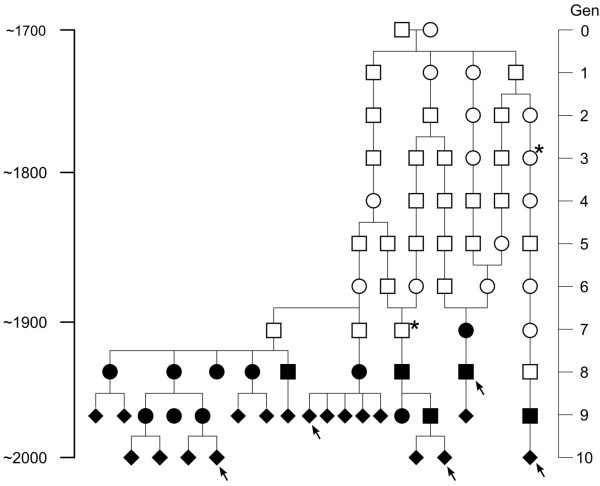
**Pedigree illustrating the results from the genealogical investigation performed in the 19 Swedish p.R518X index families.** The pedigree includes ascertained p.R518X mutation-carriers from five index families (n = 33, filled symbols, including two JLNS cases) connected by an ancestor couple born ~10 generations previously in the early 18^th^ century (1702/1703). Index cases are indicated by arrows. Mutation-carriers in the most recent generation are depicted as filled diamonds, and JLNS genotype not specified, in order to preserve the anonymity of cases. The male (square) marked with an asterisk in generation 7 was married to a descendant of the female (circle), also marked with an asterisk, in generation 3. This alternative route of possible inheritance of the p.R518X mutation was omitted for legibility.

Genealogical investigation in the 19 p.R518X index families revealed several genealogical interconnections, whereof a couple born 1702/1703 in the Pite River Valley region connected five index families over 10 generations and approximately 300 years (Figure 3).

**Figure 4:**
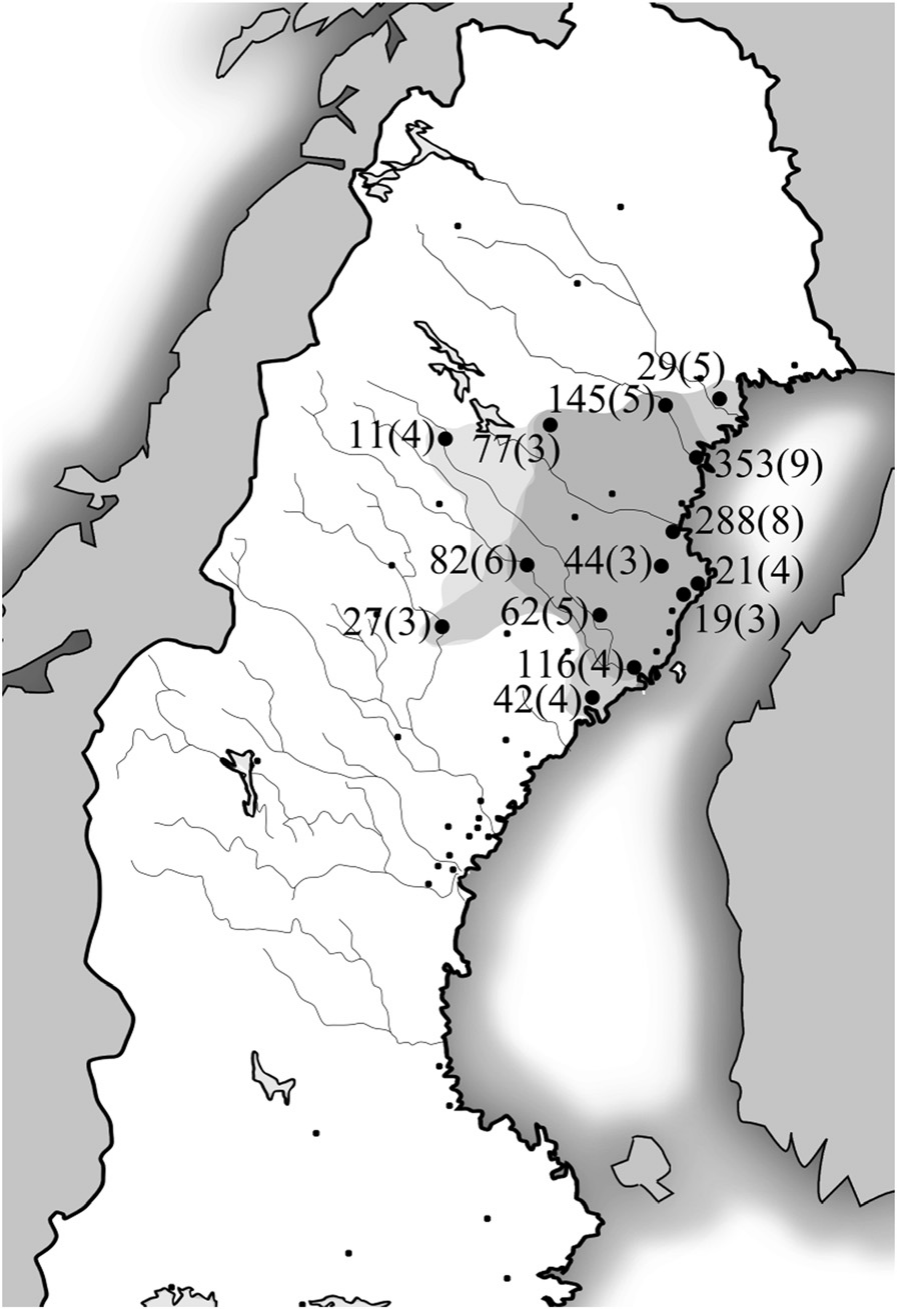
**Map of northern Sweden illustrating the clustering of birth places (dots) of p.R518X ancestors born between 1650 and 1950.** Bold dots represent parishes where ancestors from ≥3 index families co-resided during the same time-period (1650-1749, 1750-1849 and/or 1850-1949). For each time-period the area delimited by the bold dots was shaded, resulting in a darker colour in the region with highest concentration of p.R518X ancestors over time. The number of p.R518X ancestors born in each parish (whereof 93% born between 1650 and 1850) is given, followed by the maximum number of co-residing families during a single time-period (in parenthesis).

Analysis of the birth places of all identified p.R518X ancestors born between 1650 and 1950 (n = 1929) revealed a northern origin in 88% (n = 1701) and geographical clustering, over time, in the upper northern region with focus around the coastal area where the Pite River accesses the Gulf of Bothnia (Figure 4).

**Figure 5:**
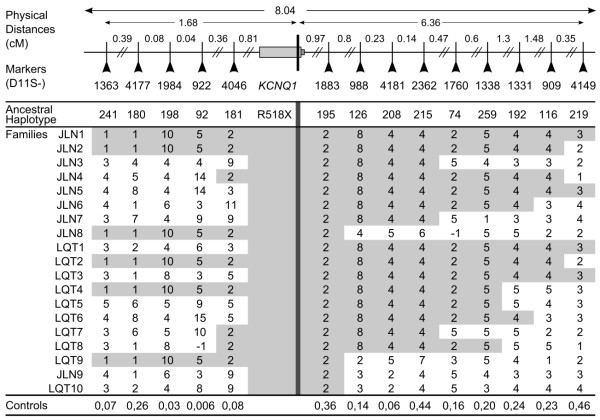
**The ancestral haplotype (x-axis) of 19 p.R518X index families (y-axis) was reconstructed, identifying 17 founder families (families JLN1-8, LQT1-9).** An overview of the 14 analysed markers and their locations is given above. The C-terminal location of the p.R518X mutation in the *KCNQ1* gene is indicated by the vertical black line. Shared alleles (4-14, median 7) are shaded in grey. The proportion of the founder alleles found in 168 control chromosomes is given for each marker (bottom). Families JLN9 and LQT10 shared only one of the downstream mutation-associated markers adjacent to the *KCNQ1* gene, but between themselves shared six markers spanning over the *KCNQ1* gene, indicating that these two families are related to each other.

Analysis of microsatellite markers in the 19 p.R518X index families (with haplotype data available from two separate generations in 15/19 families, in total 36 mutation-carriers) and 168 control chromosomes revealed a shared haplotype of uncommon alleles (control frequency 0.006-0.46, median 0.18) over 4-14 marker loci (median seven) for 17/19 families (Figure 5). Two probands (JLN9 and LQT10) did not share the founder haplotype but between themselves shared six markers spanning over the *KCNQ1* gene. The two families that did not share the founder haplotype (JLN9 and LQT10) included only two ascertained mutation-carriers (the probands). Additionally, one parent of a homozygous p.R518X proband (JLN3) had a third, separate, haplotype. Consequently, in total 98/101 included cases pertained to the founder population.

### Mutation age and prevalence estimate

The age of the identified founder haplotype was estimated to 28 generations (95% CI 19;41) assuming a mutation rate of 10^-6^, by ESTIAGE computer software. Estimates were not affected by type of mutation model used and only slightly by mutation rate (Table 2). The estimate corresponds to a mutation age of 700 years (95% CI 475-1025), when assuming 25 years per average generation. By DMLE computer software, the sampled probands with a shared haplotype (n = 17) were estimated to account for 2-4% of the entire p.R518X population (proportion of population sampled 0.02-0.04), for growth rates between 25-27% and mutation ages between 26-29 generations, with a best fit approximating 0.03. The estimate predicts between 425-850 p.R518X probands of founder descent, corresponding to a rough p.R518X mutation-carrier prevalence of ~1: 2000-4000 in Sweden (population ~9.5 × 10^6^, Table 3).

**Table 2:** ESTIAGE mutation age estimates results, in generations including 95% confidence intervals

**Mutation rate**	**Mutation model**	
	**Stepwise**	**Equal**
10^-4^	27 (18;41)	27 (18;41)
10^-6^	28 (19;41)	28 (19;41)

**Table 3:** Estimation of proportion of population sampled and corresponding prevalence estimates for the p.R518X founder mutation

**Probands sharing haplotype**	**Mutation age-span**^**a**^	**Population growth rates,%**	**Proportion sampled**	**Best fit**	**Estimated probands**	**Estimated prevalence**^**b**^
17	26-29	25-27	0.02-0.04	0.03	425-850	~1:2-4000

Estimates calculated using the DMLE computer software (http://www.dmle.org).

^a^ In generations.

^b^ Calculated as: the Swedish population size/(estimated probands × [identified mutation-carriers in founder families/probands]), i.e.:

(lower bound) 9.5 × 10^6^/ (425 × [99/17]) = 1:3838.

(upper bound) 9.5 × 10^6^/ (850 × [99/17]) = 1:1919.

## Discussion

In this study, we identified 101 Swedish cases (15 JLNS, 86 LQTS) in 19 index families segregating the R518X/*KCNQ1* mutation, and revealed that the common occurrence of this specific mutation in the Swedish population is related to founder effects.

### A benign phenotype and remaining variability

While the p.R518X-associated phenotype in JLNS cases was expectedly severe (resulting from a near-complete loss of Kv7.1 function), an unexpectedly benign phenotype was seen in LQTS cases. When in the heterozygous form, the p.R518X nonsense mutation has been shown to cause Kv7.1 haploinsufficiency, in vitro [16]. As for p.R518X, it is common for heterozygous nonsense mutations to present with relatively mild phenotypes, as the resultant protein products cannot co-assemble with wild type subunits and therefore seldom cause dominant-negative effects [17]. However, as compared to 169 heterozygous carriers of *KCNQ1* mutations causing haploinsufficiency, described by Moss *et al*[18], the clinical phenotype of the p.R518X founder heterozygotes still appear to be less severe (aborted cardiac arrest 3% vs. 0, sudden death 2.4% vs. 1.2%). The annual incidence rate of life-threatening cardiac events before therapy in the p.R518X founder heterozygotes is comparable to that of another unexpectedly benign Swedish founder population (0.04% vs. 0.05%), segregating the dominant negative p.Y111C mutation [5,6]. The benign phenotypes of the LQTS river valley populations in Sweden remain unexplained.

The popular notion that founder mutations would by their nature be benign is negated by the severe phenotype of the A341V/*KCNQ1* mutation that segregates within a South African founder population, and is associated with a staggering 30% cumulative incidence of life-threatening cardiac events [19]. As in the case with the Boer progeny [20], a substantial intra-familial phenotypic variability regarding clinical phenotype remained in the p.R518X founder population, and the symptomatic phenotype in p.R518X heterozygotes did not correlate with gender, heart rate nor, surprisingly, QTc.

### The commonly occurring p.R518X mutation

In the international context p.R518X is commonly described as a hotspot mutation, reported as a common cause of JLNS [8,9,16], as well as one of the five most common mutations in Northern American LQTS probands [2]. In Sweden, the p.R518X mutation has previously been revealed as the major cause of JLNS, contributing to the JLNS genotype in 9/12 identified JLNS index families with ascertained genotype (12/24 alleles) [4]. The importance of the p.R518X allele also with regards to the Swedish LQTS mutation spectrum was indicated by the finding that p.R518X was the second most common mutation identified in 200 Swedish index cases referred for LQTS diagnostics at the laboratory of Clinical Genetics, Umeå University Hospital, between 2006 and 2009 [3]. The cohort included LQTS index cases (excluding JLNS probands) originating from all six Swedish health care regions, whereof 78% from without the northern region. Together with p.Y111C (the most commonly identified mutation) these two mutations accounted for over 25% of index cases with ascertained genotype (n = 102) [3].

**Figure 6:**
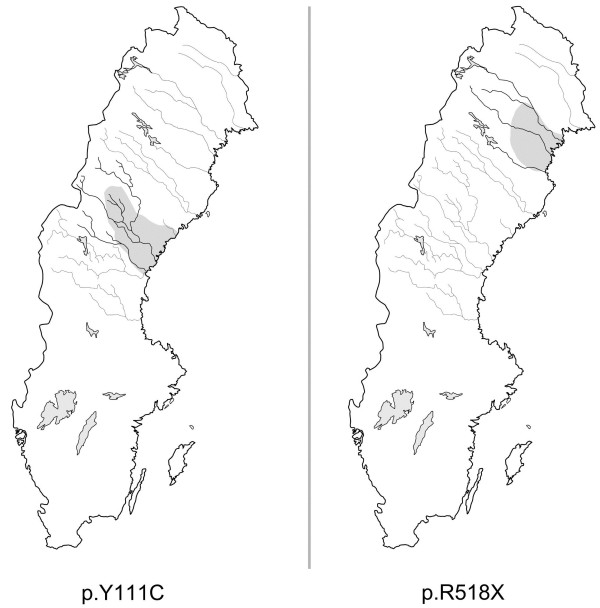
**The geographic distribution of the two northern river valley regions identified as the origins of the Swedish LQTS founder mutations Y111C/*KCNQ1 *** **(left) and R518X/*KCNQ1 *** **(right).**

While the majority of the Swedish p.R518X JLNS and LQTS probands have now been identified as pertaining to a founder population, the additional finding of two p.R518X probands, as well as one parent to a homozygous p.R518X case, lacking the founder haplotype supports the occurrence of p.R518X also as a hotspot mutation in the Swedish setting. In parallel, in our neighbouring country Norway, with which we share cultural and linguistic similarities, geographical proximity, history (political union 1814-1905), as well as marked similarities regarding the JLNS mutation spectra [4], the p.R518X mutation also occurs both as a hotspot and a founder mutation [21]. These findings are in no way controversial, as the frequent occurrence of a particular mutation (which in this case also is associated with a relatively benign phenotype) would intuitively make it more likely to happen to partake in a founder effect given the right population-developmental circumstances. As we have shown for the Y111C/*KCNQ1* founder mutation [5], congruent genealogical and haplotype data including mutation dating placed the founding of the p.R518X population in a northern river valley region around the 14^th^ century, coinciding with the early phase of a royal initiative to populate the upper northern regions of Sweden [22]. The geographical location of the separate river valley regions of the Swedish founder mutations is presented in Figure 6. As for p.Y111C, the combination of a low number of original founders, a slow initial population growth, a geographical isolation of the river valleys, and a preference to marry within the river valley region, probably promoted the enrichment of founder alleles in the Swedish population [5,23]. As a curiosity, in spite of the common occurrence of the p.R518X and p.Y111C mutations in the northern region and the high frequency of JLNS cases, no case with p.R518X/p.Y111C genotype has been identified, the reason for which remains unknown [4].

### Limitations

The major LQTS genes were not analysed in 4/10 LQTS probands, negating the possibility of identifying additional mutations contributing to phenotype in these cases.

The p.R518X prevalence estimate of ~1:2000-4000 presented in this study is based on extrapolations from genetic, genealogical and epidemiological data, and as such should be interpreted with caution. Being derived from data on the founder population the estimates do not include calculations regarding p.R518X cases secondary to hotspot effects (risk of underestimation). Also, regional differences in prevalence with regards to distribution of mutation-carriers are to be expected (i.e. higher prevalence in the northern region and the major urban regions in the south that have received the majority of the 20-21^th^ century migration). The p.R518X prevalence estimate of ~1:2000-4000 is supported by the high frequency of both founder (n = 14) and non-founder JLNS cases (n = 1) with p.R518X mutations identified in the population, the high contribution of the specific p.R518X allele to the Swedish JLNS mutation spectrum [4], as well as congruent prevalence estimates in the Norwegian population [9].

## Conclusions

The common occurrence of the p.R518X mutation among Swedish probands with recessive and dominant type LQTS is mainly secondary to a founder effect. Our findings suggest a high prevalence of the p.R518X founder mutation in the Swedish population. In the clinical setting, due to the low penetrance of clinically identifiable markers in p.R518X heterozygotes, molecular genetics diagnosis of probands and cascade-screening of first-degree relatives remains imperative in order to identify individuals at risk of developing preventable arrhythmia.

## Competing interests

There are no competing interests to declare.

## Authors’ contributions

AW designed the study, participated in the data collection including interviews, performed all analyses on clinical and molecular genetics data including basic statistical analyses, participated in the advanced statistical analyses, interpreted all data, designed tables and figures, drafted the manuscript and wrote the final manuscript. ELS participated in conceiving of the study, data collection and supervised the molecular genetics studies. CN carried out and interpreted the genealogical studies. UBD supervised and participated in the genealogical studies and participated in data collection. JP participated in the design of the study and performed the advanced statistical analyses. SJ participated in conceiving of the study as well as data collection. AR participated in conceiving of the study, participated in its design and coordination, as well as supervised and participated in data collection. All authors participated in the critical revision of the manuscript, as well as read and approved the final manuscript.

## Authors’ information

AW is a medical doctor in Umeå, Sweden. In 2012 she published her thesis on LQTS founder effects and associated cardiac phenotypes in the Swedish population. AR is a pediatric cardiologist and the principle investigator of the LQTS research group. Together with clinical geneticist ELS, cardiologist SJ and biomedical analyst UBD she founded the LQTS Family Clinic in Umeå, Sweden, where LQTS families including several generations of carriers have been counselled and treated in a multi-disciplinary setting since 2005.

## Pre-publication history

The pre-publication history for this paper can be accessed here:

http://www.biomedcentral.com/1471-2261/14/22/prepub
